# Subjective and objective assessment of physical activity in multiple sclerosis and their relation to health-related quality of life

**DOI:** 10.1186/s12883-016-0783-0

**Published:** 2017-01-13

**Authors:** Theresa Krüger, Janina R. Behrens, Anuschka Grobelny, Karen Otte, Sebastian Mansow-Model, Bastian Kayser, Judith Bellmann-Strobl, Alexander U. Brandt, Friedemann Paul, Tanja Schmitz-Hübsch

**Affiliations:** 1 NeuroCure Clinical Research Center, Clinical Neuroimmunology Group, Charité – Universitätsmedizin Berlin, Charitéplatz 1, 10117 Berlin, Germany; 2 Department of Neurology, Charité – Universitätsmedizin Berlin, Charitéplatz 1, 10117 Berlin, Germany; 3 Experimental and Clinical Research Center, Charité – Universitätsmedizin Berlin and Max Delbrück Center for Molecular Medicine, Lindenberger Weg 80, 13125 Berlin, Germany; 4 Motognosis UG, Schönhauser Allee 177, 10119 Berlin, Germany

**Keywords:** Physical activity, Accelerometry, IPAQ, Multiple sclerosis, Quality of life

## Abstract

**Background:**

Physical activity (PA) is frequently restricted in people with multiple sclerosis (PwMS) and aiming to enhance PA is considered beneficial in this population. We here aimed to explore two standard methods (subjective plus objective) to assess PA reduction in PwMS and to describe the relation of PA to health-related quality of life (hrQoL).

**Methods:**

PA was objectively measured over a 7-day period in 26 PwMS (EDSS 1.5–6.0) and 30 matched healthy controls (HC) using SenseWear mini® armband (SWAmini) and reported as step count, mean total and activity related energy expenditure (EE) as well as time spent in PA of different intensities. Measures of EE were also derived from self-assessment with IPAQ (International Physical Activity Questionnaire) long version, which additionally yielded information on the context of PA and a classification into subjects’ PA levels. To explore the convergence between both types of assessment, IPAQ categories (low, moderate, high) were related to selected PA parameters from objective assessment using ANOVA. Group differences and associated effect sizes for all PA parameters as well as their relation to clinical and hrQoL measures were determined.

**Results:**

Both, SWAmini and IPAQ assessment, captured differences in PA between PwMS and HC. IPAQ categories fit well with common cut-offs for step count (*p* = 0.002) and mean METs (*p* = 0.004) to determine PA levels with objective devices. Correlations between specifically matched pairs of IPAQ and SWAmini parameters ranged between r .288 and r .507. Concerning hrQoL, the lower limb mobility subscore was related to four PA measures, while a relation with patients’ report of general contentment was only seen for one.

**Conclusions:**

Both methods of assessment seem applicable in PwMS and able to describe reductions in daily PA at group level. Whether they can be used to track individual effects of interventions to enhance PA levels needs further exploration. The relation of PA measures with hrQoL seen with lower limb mobility suggests lower limb function not only as a major target for intervention to increase PA but also as a possible surrogate for PA changes.

**Electronic supplementary material:**

The online version of this article (doi:10.1186/s12883-016-0783-0) contains supplementary material, which is available to authorized users.

## Background

A low level of physical activity (PA) is a known risk factor for health outcomes and effects on disease outcomes have been described in several conditions [1–3].

Also in people with multiple sclerosis (PwMS), aiming to enhance PA is considered beneficial [4]. Like in other chronic disorders, PA assessment may serve as a marker of disability status [5] with different aims: on the one hand to select individuals that may benefit from intervention and to track effects of behavioral interventions and on the other hand to investigate how and which functional changes translate into real-life activity [6].

We here use the term PA according to the classic definition as “all muscle activity exerted by an individual resulting in energy expenditure (EE) above resting EE” [7]. Thus, the intensity of PA is usually expressed in multitudes of individual resting EE, designated as metabolic equivalent of task (MET, unit of work/ kg body weight/ hour), with estimates assigned to different types of PA [8–10]. According to their assigned MET, PA is usually categorized as low, moderate or vigorous activity (LPA, MPA, VPA). In contrast, daily step count or time spent walking specifically refer to locomotor activity which can take on different intensities, e.g. depending on walking speed or inclination. General levels of PA are most often described as step count, total daily EE, activity related EE or time of the day spent active/ inactive as defined by MET thresholds.

The different methods used to assess PA capture different aspects and have their specific limitations [10–13]. The International Physical Activity Questionnaire (IPAQ) [14] is a standardized self-rating questionnaire based on recall over the previous week.

Several portable activity monitors have become available for objective PA assessment [3, 10, 12, 15–17]. Accuracy of the multi-sensor device SenseWear Pro was acceptable in PwMS [18] against indirect calorimetry as standard, while - to our knowledge - the successor SWAmini with reportedly improved performance [19–21] has not been applied in PwMS. We therefore aim to explore its applicability in this population and convergence of results with those obtained from subjective assessment (IPAQ long version). From previous reports we expect lower levels of daily PA to be associated with more severe symptoms of MS and reduced health-related quality of life (hrQoL). By correlation of PA measures with an MS-specific quality of life questionnaire, we aim to define which aspects of MS-related functional impairment relate most closely to decline in daily PA level in PwMS.

## Methods

### Study participants

Study participants were enrolled at a university MS referral center. Inclusion criteria for all participants were: age 18–65 years, no cardiovascular disease, orthopedic or other conditions thought to affect motor performance or daily activity and additionally for healthy controls (HC) without neurological diagnosis: no impairment of gait or balance evident at testing. Further inclusion criteria for PwMS were: MS diagnosis according to McDonald Criteria 2010 [22], EDSS between 0 and 6, no relapses for at least 30 days prior to study visit, no other neurologic comorbidity. The study was approved by the local ethics committee of the Charité - Universitätsmedizin Berlin (EA1/321/14) and conducted in conformity with the Declaration of Helsinki in its currently applicable form. All participants gave written informed consent.

### Objective assessment of daily PA with SWAmini

We used the physical activity monitor SenseWear® Armband and Software Development Kit Version 8.1.1.30 (SenseWear Model Mini, MF-SW; BodyMedia®, Inc. Pittsburgh, Pennsylvania, USA). Subjects were instructed to wear the SWAmini over 7 days (including the weekend) throughout the day and to take the sensor off during night rest and for any water activity. The monitor was placed at the middle of the triceps brachii muscle (left arm) according to the user manual [23]. The device reports individual wearing times that served to prove subjects’ compliance.

SWAmini reports physical activity as accelerometrically derived step count and as estimates of individual EE from 1-min epochs of recording based on algorithmic integration of multi-sensor data incorporating subject’s age, sex and body size. EE is reported as total MET per recording time (mean METs) and activity related EE (active METs). One MET represents estimated resting EE and by convention equals 1 kcal/ kg body weight/h. The intensity of PA within each epoch is classified according to MET cut-offs of 1.5–3 for light PA (LPA), MET 3 – 6 for moderate PA (MPA), MET 6–9 for vigorous PA (VPA) and MET > 9 for very vigorous PA (VVPA). Duration of PA per intensity is reported in min/hour, calculated as SWAmini output per day divided by individual daily wearing time, MPA and VPA values were combined into MVPA for some analyses. A list of parameters is available as Additional file 1: Table S1.

To account for possible difference in PA behavior between weekdays and weekend, a weighted mean was calculated as follows:

$$ \frac{\left(\mathrm{parameter}\ \mathrm{mean}\ \mathrm{weekend}\times 2\right)+\left(\mathrm{parameter}\ \mathrm{mean}\ \mathrm{weekday}\times 5\right)}{7} $$

For comparison with IPAQ data, activity-related EE was given in MET*min/day calculated as SWAmini mean active METs times duration of all MET > 3 activity per day. Similarly, SWAmini MPA and VPA duration in min/hour were transformed into min/day by multiplication with the mean daily hours of wearing time.

### Subjective assessment of daily PA with IPAQ long version

We used the German (Austrian) translation of the IPAQ long version [14] that reports the amount and intensity of patients’ PA by recall of the past 7 days. The questionnaire was applied in paper-pencil form after the week of SWAmini recording and thus, IPAQ data refer to the same time span as SWAmini recordings.

After application of data cleaning rules according to the scoring manual [24] IPAQ results were coded, first, as time per day spent in PA of different intensity, i.e. time spent walking (IPAQ Walking duration), time spent in moderate (IPAQ MPA duration) or vigorous PA (IPAQ VPA duration), that can be summed to total duration of daily PA (IPAQ Total duration). Data are usually presented as min/week that we transformed into min/day for comparison with SWAmini data. As the MET-value of 3.3 assigned for walking in the IPAQ [14] is above the MET- threshold for MPA in the SWAmini, we computed a combined parameter from IPAQ Walking duration + IPAQ MPA duration for comparison. Second, results were rendered as estimates of active EE by multiplication of duration of activity with predefined MET values given in MET*min/week that we transformed into MET*min/day for comparison with SWAmini. Third, both the duration and intensity of PA were coded separately in four ADL domains: work, active transportation, domestic/yard and leisure/sports that were reported as EE per domain in % of total active EE. Fourth, we applied the scoring rules to classify individuals into the IPAQ categories of low, moderate and high PA level.

In sum, IPAQ long form yielded three main outcomes: 1) the activity related EE as well as time spent for walking, MPA and VPA, 2) the distribution of EE in the four different ADL domains and 3) a three-step classification of PA level. In addition, we derived information on occupational status out of IPAQ results.

### Clinical severity and health-related Quality of Life

In PwMS, a neurological examination with the Expanded Disability Status Scale (EDSS) scoring [25] was performed prior to SWAmini assessment. The Hamburg Quality of Life Questionnaire in Multiple Sclerosis (HAQUAMS version 10.0) was applied in paper form in its German version (HALEMS) [26, 27]. It consists of 44 items, 28 of which contribute to the total score between 1 (unimpaired) and 5 (very much impaired) as the mean of the following six subscores: fatigue, thinking, mobility lower limb, mobility upper limb, communication and mood. We added a sensory (items 4 and 5) and bladder/bowel (items 26–28) subscore, as we assumed these may impact on subjects’ daily PA. As a general measure of overall contentment in life – irrespective of any disease specific impairment – we included item 43 (score 1–5) into analysis.

### Statistical analysis

Differences between groups (HC and PwMS) regarding sex and occupational status were calculated using the *χ*2-test while independent sample t-tests were applied with regard to age, height, weight and BMI and SWAmini parameters. Differences of SWAmini parameter means between weekdays and weekends were explored by paired *t*-test, separately for each group. Group differences for SWAmini parameters were also expressed as effect sizes (Cohen’s d) calculated as mean between-group difference divided by the standard deviation in HC. Between-group differences regarding IPAQ parameters of duration and EE were explored using Mann–Whitney-U-tests. The proportions of IPAQ Total EE spent in the four domains work, transportation, domestic and leisure as well as the distribution of subjects into IPAQ categories of low, moderate and high PA level was compared between groups with χ2-test.

We used Spearman rank correlation for IPAQ data, EDSS and HAQUAMS total and subscores. To relate SWAmini parameters step count and mean METs to the IPAQ categories of low, moderate and high PA level, we applied ANOVA with Bonferroni post-hoc testing, while EDSS scores were related to the IPAQ categories with Jonckheere-Terpstra-Test.

All analyses relating SWAmini to IPAQ as well as inter-correlations of SWAmini parameters (presented as Additional files 1, 2, 3 and 4) were performed for the whole group, while correlations with EDSS and HAQUAMS were only analyzed in PwMS.

Statistical analysis was performed with SPSS version 22 (IBM, Armonk, NY, USA). All tests were two-tailed, significance was assumed when *p* < 0.05. No alpha-error correction for multiple testing was applied in this exploratory study.

## Results

### Study cohort and data cleaning

From November 2014 to August 2015, 29 PwMS and 30 gender- and age-matched HC were included into the study. Three PwMS were excluded because of incorrect use of the SWAmini (2) or missing SWAmini-data (1). IPAQ data were missing in one HC, and data of one PwMS were excluded in the data cleaning process (>960 min of daily activity time). Truncation of IPAQ active time according to the IPAQ manual had to be applied in 5 subjects (3 HC and 2 PwMS). Data in HAQUAMS item 43 (contentment) were missing in one PwMS. Thus, analyses refer to 26 PwMS and 30 HC (Table 1), while analyses comprising IPAQ data refer to 26 PwMS and 29 HC. A higher rate of unemployment was noted among PwMS that seemed related to higher EDSS (Table 1, Additional file 3: Figure S1).

**Table 1:** Cohort overview – demographic and clinical data per subject group

		HC	PwMS	*p*-value
Subjects	N	30	26	
	Relapsing MS		18	
	Progressive MS		8 (7 SPMS; 1 PPMS)	
Sex	Male /female	10 /20	10 /16	.783
Occupational status	without employment/ working	6/23	14/12	.013*
Age (years)	Mean (±SD)	49.7 (±8.3)	50.9 (±5.2)	.522
BMI	Mean (±SD)	25.3 (±3.9)	26.0 (±3.5)	.474
Height (m)	Mean (±SD)	1.70 (±7.4)	1.71 (±6.6)	.398
Weight (kg)	Mean (±SD)	73.8 (±13.7)	76.8 (±12.9)	.588
EDSS	Median (Min-Max)	.	4.0 (1.5–6.0)	

Independent sample *t*-test was used for between-group comparison of age, BMI, weight and height and χ2-test was used to compare sex and occupational status. Please note that IPAQ-data and thus occupational status from 1 HC is missing. *denotes significance at level <0.05

### SWAmini: objectively assessed daily PA

Only small differences between parameter means from weekend and weekday were seen in HC with 0.15 lower active METs (*p* = 0.049) on weekends. No difference was seen for any parameter in PwMS between weekend/weekday. Therefore, all further SWAmini analyses refer to a weighted mean.

Between-group differences - that is, HC performing better than PwMS - were observed in all parameters except light PA (*p* < 0.01, Table 2). Highest effect sizes (Cohen’s d ≥ 0.85) were seen with step count, mean and active METs, while other parameters yielded effect sizes between 0.63 and 0.75 (Table 2).

**Table 2:** Amount and intensity of daily physical activity in HC and PwMS assessed with wearable device (SWAmini) reported as weighted mean (± SD) and subjective assessment with IPAQ long version reported as median (interquartile range) from a 7-day period

		Healthy controls	PwMS	*p*-value	Cohen’s d
		*N* = 30	*N* = 26		
Objective (SWAmini)	Step count (steps/hour)	563.45 (±155.90)	430.66 (±171.68)	**.004**	0.85
	Mean METs (mean/day)	1.77 (±.28)	1.53 (±.16)	**<.001**	0.86
	Active METs (mean/day)	4.41 (±.59)	3.90 (±.46)	**.001**	0.86
	Total EE (kcal/hour)	387.40 (±237.40)	214.08 (±194.20)	**.004**	0.73
	Active EE (kcal/hour)	118.13 (±110.65)	37.89 (±41.73)	**.001**	0.75
	Active EE (MET*min/day)	410.79 (±257.07)	217.70 (±116.19)	**.001**	0.75
	LPA (min/hour)	2.98 (±2.47)	3.90 (±3.27)	.249	0.37
	MPA (min/hour)	5.38 (±2.63)	3.73 (±1.80)	**.008**	0.63
	MVPA (min/hour)	6.23 (±3.21)	3.91 (±1.93)	**.002**	0.72
	VPA (min/hour)	.844 (±.927) *3	.176 (±.403) *6	**.001**	0.72
	VVPA (min/hour)	.148 (±.426) *21	.001 (±.002) *25	.084	0.35
	Full days worn (days/period)	7.23 (±1.36)	7.81 (±1.77)	.220	-
	Mean daily wearing time (hours/day)	15.38 (±1.73)	14.29 (±1.58)	.017	-
Self-perceived (IPAQ)	Walking duration (min/day)	27.86 (45.71) *2	34.29 (71.14) *2	.664	
	MPA duration (min/day)	51.43 (91.79) *6	0.00 (53.57) *14	**.010**	
	MVPA duration (min/day)	68.19 (101.79) *6	0.00 (60.00) *13	**.016**	
	VPA duration (min/day)	2.86 (25.71) *14	0.00 (17.14) *17	.259	
	Total duration (min/day)	108.57 (120.36)	81.43 (135.00)	.327	
	Total EE (MET*min/day)	516.57 (502.04)	501.14 (596.44)	.440	
	Work EE (mean % of total EE and interquartile range)	19.12% (31.22) *8, 6 unemployed	12.66% (17.34) *5, 14 unemployed	.089	
	Transportation EE (mean % of total EE and interquartile range)	34.41% (43.38) *2	28.42% (40.53) *2		
	Domestic EE (mean % of total EE and interquartile range)	24.93% (22.43) *2	28.34% (33.76) *3		
	Leisure EE (mean % of total EE and interquartile range)	21.54% (28.70) *4	30.58% (36.59) *2		
	IPAQ category (n and % of group):			.150	
	Low	5 (17.2%)	10 (40.0%)		
	Moderate	21 (72.4%)	14 (56.0%)		
	High	3 (10.3%)	1 (4.0%)		

Subjects were instructed to wear SWAmini during the waking day excluding water activities. *P*-values for between-group differences of SWAmini parameters refer to t-tests. IPAQ-data (comprising 29 HC and 25 PwMS) were compared between groups using Mann–Whitney-*U*-test, except for *χ*2-test used to compare the distribution of subjects into different IPAQ physical activity levels and the percentages of total EE spent in different activity domains. Please note that percentage values in IPAQ work EE were set to a value of 0 for unemployed subjects. Numbers of subjects per group that were not assigned any activity for MPA/VPA/VVPA are given with asterisk; these individuals were assigned a value of 0 for the calculation of means

**Fig. 1:**
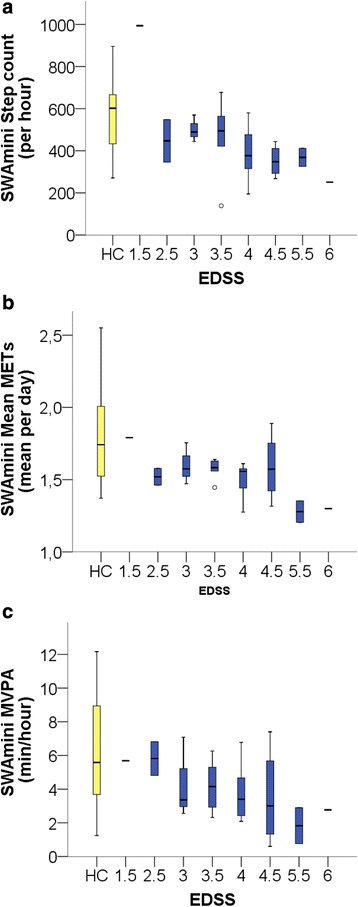
Selected activity parameters from SWAmini related to EDSS score. **a** Steps per day, **b** Mean METs and **c** MVPA. Results of HCs are depicted in *yellow* for reference, all PwMS results are depicted in *dark blue*. *Lines* refer to median, *boxes* to interquartile range and whiskers to maximum and minimum excluding outliers (outlier defined as > 1.5 IQR of upper or lower quartile)

As expected from the observed between-group differences, the correlations of SWAmini parameters with EDSS were not significant for time spent in light activities, but pointed to a decrease of higher intensity PA with higher EDSS (Fig. 1), reflected in decreased step count (r −0.534, *p* = 0.005), mean METs (r −0.411, *p* = 0.037), MPA and MVPA (r −0.477, *p* = 0.014 and r −0.451, *p* = 0.021). The lack of correlation with VPA and active METs (r −0.327, *p* = 0.103 and r −0.175, *p* = 0.394) is likely due to the generally low amount of VPA in this group with no VPA recorded in 6 of 26 PwMS (Table 2).

### IPAQ: subjectively assessed daily PA

Concerning the proportional distribution of IPAQ total EE into ADL domains, a trend for group differences was noted (*p* = 0.089, Table 2) with a tendency of PwMS to spend a smaller part of their daily total EE in active transportation/work but a larger part in leisure and domestic activities (Table 2). This suggests active transportation as a work-related activity.

**Fig. 2:**
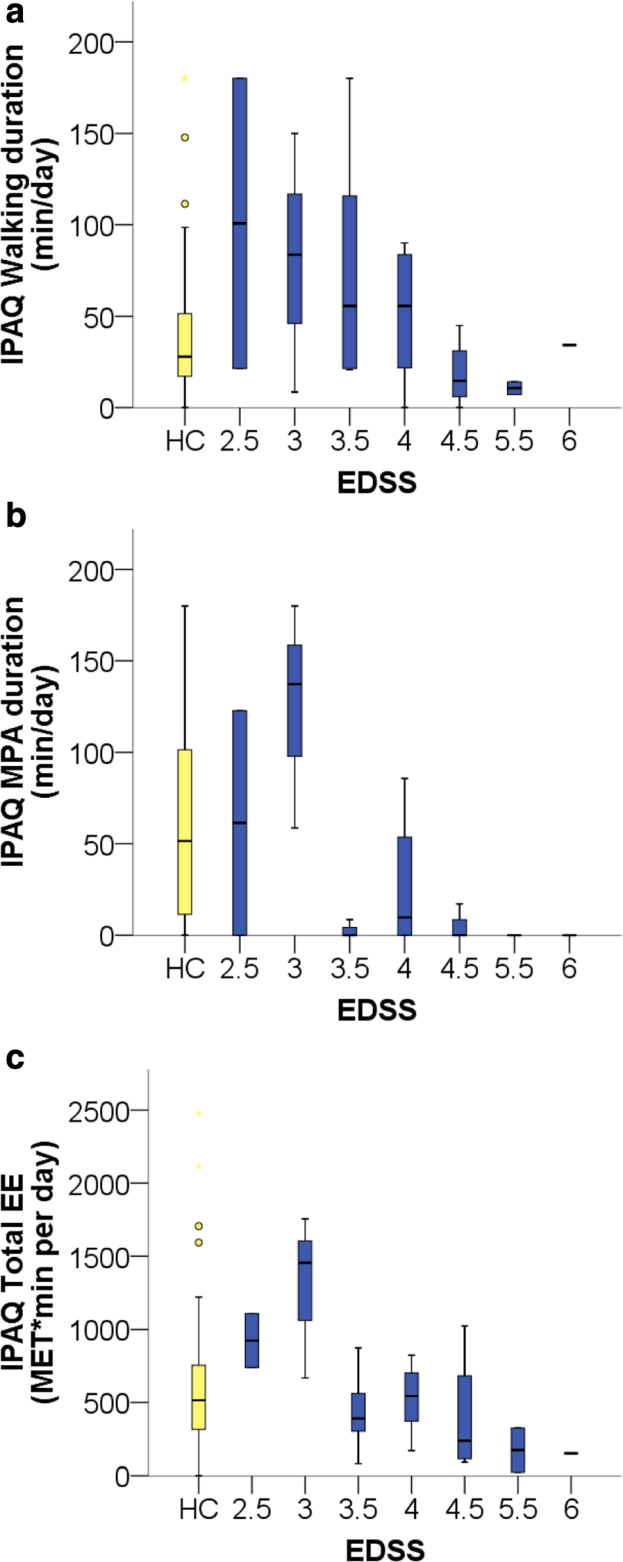
Selected activity parameters from IPAQ related to EDSS score. **a** Walking duration, **b** MPA duration and **c** Total EE. Results of HCs are depicted in *yellow* for reference, all PwMS results are depicted in *blue. Lines*, *boxes*, *whiskers* as in Fig. 2. Please note that the one subject with EDSS 1.5 was excluded in the data cleaning process according to the IPAQ manual

Second, we explored group differences in total durations of PA irrespective of their contextual domain (Walking, MPA, VPA, MVPA and Total PA duration). PwMS reported less time spent in moderate PA (MWU; MPA *p* = 0.010 and MVPA *p* = 0.016), while no significant differences were seen for walking, vigorous PA, total PA duration as well as total activity-related EE (Table 2). It should be noted for interpretation that as with SWAmini, a considerable number of subjects reported no vigorous (14 HC and 17 PwMS) or even moderate activities (6 HC and 14 PwMS). Similar to SWAmini, reduced PA in PwMS was associated with higher EDSS scores (Fig. 2) for IPAQ Total duration (r −0.752, *p* < 0.001), IPAQ Total EE (r −0.572, *p* = 0.003), IPAQ Walking duration (r −0.462; *p* = 0.020,) MPA duration (r −0.469, *p* = 0.018), VPA duration and MVPA duration (r −0.428, *p* = 0.033 and r −0.424, *p* = 0.035).

**Fig. 3:**
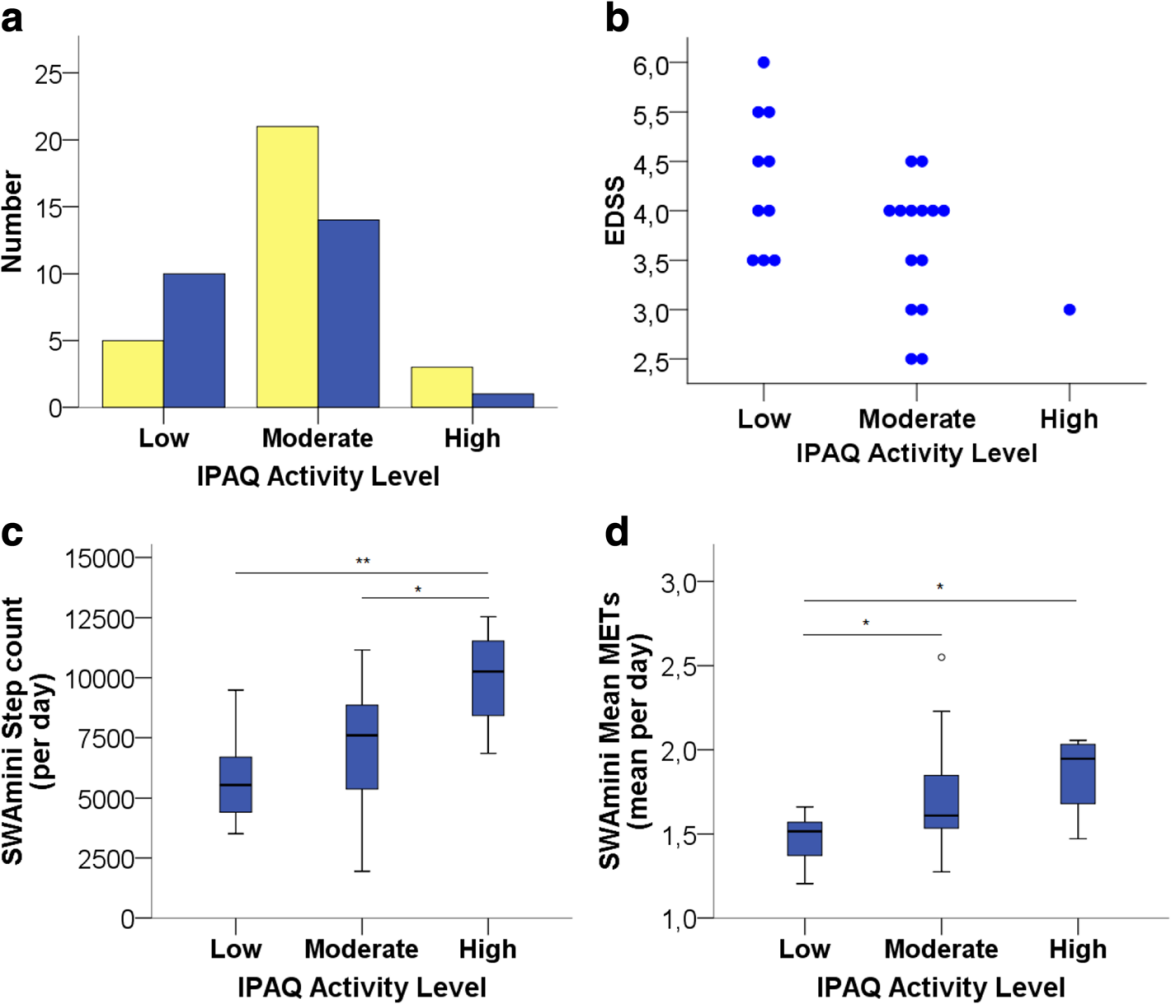
IPAQ categories of physical activity level. **a** Frequency per category and group, **b** Relation with EDSS, **c** Relation with SWAmini Step count and **d** Relation with SWAmini Mean METs. HCs are depicted in *yellow*, PwMS in *blue*. Please note that **c** and **d** include data from all subjects, PwMS and HC

Third, we compared the distribution of subjects into the three IPAQ categories of low – moderate – high PA level between groups (Fig. 3a). There was a tendency of HC to be more frequently classified as moderate or high PA level and less frequently as low PA level compared to PwMS (X^2^ test, *p* = 0.150, Fig. 3a). EDSS scores differed across the IPAQ PA levels with higher EDSS scores in PwMS assigned as low activity level and low EDSS of 3 in the one highly active MS subject (Jonckheere-Terpstra test; *p* = 0.027; Fig. 3b).

### SWAmini and IPAQ: associations between subjectively and objectively assessed daily PA

For testing convergence of both methods we correlated three matching pairs of PA parameters (Additional file 4: Figure S2). The combined parameter moderate PA and walking duration from the IPAQ showed a remarkable association with moderate PA assessed with SWAmini (IPAQ MPA + Walking duration and SWAmini MPA Spearman r .507, *p* < 0.001); subjectively assessed total EE was associated with respective active EE assessed with SWAmini (IPAQ Total EE and SWAmini active EE r .336, *p* = 0.013) and subjective duration of vigorous activities duration was related to the corresponding objectively assessed parameter (IPAQ VPA duration and SWAmini VPA duration r .288, *p* = 0.035). Interestingly, of the nine subjects with objectively no vigorous activities (3 HC, 6 PwMS), eight (3 HC, 5 PwMS) reported also no vigorous activities with IPAQ, while vice versa, in subjects with no self-perceived vigorous PA between 0–28 min/day were recorded with SWAmini.

In the second approach, we related the three IPAQ categories of PA level to those SWAmini parameters that have been proposed as classification criteria for individual activity level: total daily step count and mean METs. As expected, both parameters increased from low to moderate to high IPAQ activity level (ANOVA *p* = 0.002 for step count per day, *p* = 0.004 for mean METs per day, Fig. 3c and d). Post-hoc analyses indicated a better differentiation between high and moderate PA level by step count and between moderate and low PA level by mean METs (Fig. 3c and d).

### HAQUAMS: the impact of MS on quality of life related to daily PA

The distribution of HAQUAMS total and subscores were found as expected from other MS cohorts of similar disease stage without major floor or ceiling effects (Tables 3 and 4). HAQUAMS total had a median of 2.20 (range from 1.20 to 3.39) and the median of the single item 43 (global contentment) was 2.00 (range from 1 to 4) with only two subjects scoring category 1 or 4, respectively. Lower EDSS scores were strongly associated with better self-perceived hrQoL in the subscores mobility lower limb (r 0.590, *p* = 0.002), mobility upper limb (r 0.479, *p* = 0.013) and global contentment (r 0.445, *p* = 0.026), but not with other subscores or HAQUAMS total.

**Table 3:** Results of HAQUAMS total and susbcores reported as median (variance) and range in 26 PwMS and their association with daily physical activity according to SWAmini parameters (Spearman correlations)

HAQUAMS parameter	Median (variance)	Min-Max	SWAmini											
			Step count per hour		Mean METs		Active METs		LPA		MPA		VPA	
			rho	*p*	rho	*p*	rho	*p*	rho	*p*	rho	*p*	rho	*P*
Fatigue	2.61 (.866)	1.00–4.25	−.136	.509	−.369	.064	−.156	.446	.350	.080	−.244	.230	−.233	.253
Thinking	2.75 (.980)	1.00–4.50	−.061	.768	−.109	.595	−.186	.364	.124	.547	−.065	.754	−.187	.361
Mobility lower limb	2.13 (.672)	1.00–3.75	**−.442**	**.024**	−.222	.275	−.064	.755	−.069	.738	**−.439**	**.025**	−.310	.123
Mobility upper limb	1.30 (.300)	1.00–3.40	−.178	.385	−.004	.985	.054	.795	−.030	.884	−.267	.188	.000	.999
Communication	2.00 (.561)	1.17–3.83	−.114	.579	−.098	.633	−.083	.689	−.040	.845	−.053	.797	−.006	.975
Mood	2.50 (.716)	1.40–4.60	−.034	.868	−.067	.745	−.202	.322	.128	.533	−.313	.120	−.128	.533
Pain/Sensory	2.00 (.600)	1.00–3.50	.145	.480	−.083	.686	−.232	.255	−.066	.749	.206	.313	**−.473**	**.015**
Bowel/Bladder	1.67 (.549)	1.00–3.33	.000	.999	−.023	.913	.099	.631	−.134	.515	−.127	.538	.090	.662
Contentment	2.00 (.427)	1.00–4.00	−.060	.776	−.026	.903	−.372	.067	−.144	.492	−.161	.442	−.365	.073
Total	2.20 (.299)	1.20–3.39	−.227	.264	−.262	.196	−.212	.298	.130	.528	−.368	.065	−.257	.205

Pain/sensory and bowel/bladder were included in addition to published HAQUAMS subscores. Contentment refers to a single-item question. Significant results at level of <0.05 are given in bold

**Table 4:** Results of HAQUAMS total and susbcores reported as median (variance) and range in 26 PwMS and their association with daily physical activity according to selected IPAQ

HAQUAMS parameter	Median (variance)	Min-Max	IPAQ									
			Duration (min/day)								EE (MET*min/day)	
			Walking		MPA		VPA		Total		Total	
			rho	*p*	rho	*p*	rho	*p*	rho	*p*	rho	*p*
Fatigue	2.61 (.866)	1.00–4.25	.190	.364	−.340	.097	−.390	.054	−.053	.803	−.150	.475
Thinking	2.75 (.980)	1.00–4.50	.133	.527	−.227	.276	−.164	.433	−.055	.793	−.016	.939
Mobility lower limb	2.13 (.672)	1.00–3.75	−.107	.610	**−.413**	**.040**	**−.441**	**.027**	−.274	.185	−.221	.289
Mobility upper limb	1.30 (.300)	1.00–3.40	−.151	.471	−.210	.315	−.171	.413	−.326	.112	−.342	.095
Communication	2.00 (.561)	1.17–3.83	−.074	.725	−.201	.336	−.289	.160	−.180	.390	−.160	.444
Mood	2.50 (.716)	1.40–4.60	−.039	.855	.087	.678	−.073	.728	.044	.836	−.028	.895
Pain/Sensory	2.00 (.600)	1.00–3.50	.344	.092	.012	.955	.099	.639	.091	.664	−.016	.939
Bowel/Bladder	1.67 (.549)	1.00–3.33	.048	.818	.132	.528	.092	.663	.226	.227	.249	.229
Contentment	2.00 (.427)	1.00–4.00	−.253	.233	−.110	.610	−.221	.299	−.360	.084	**−.489**	**.015**
Total	2.20 (.299)	1.20–3.39	−.030	.887	−.219	.294	−.294	.154	−.143	.495	−.084	.690

Pain/sensory and bowel/bladder were included in addition to published HAQUAMS subscores. Contentment refers to a single-item question. Significant results at level of <0.05 are given in bold

When we explored the relation of HAQUAMS total and subscores with the amount of daily PA from both, objective and subjective assessment, correlations of moderate magnitude were seen with HAQUAMS subscore mobility lower limb for four PA parameters: step count, moderate PA assessed objectively and subjectively as well as subjective vigorous PA (Tables 3 and 4), all pointing to higher PA levels being associated with better self-perceived lower limb function. In addition, vigorous PA assessed with SWAmini showed moderate correlation to HAQUAMS pain/sensory subscore (Tables 3 and 4). In contrast, PA parameters were not related to HAQUAMS total or global contentment, except for its moderate correlation with IPAQ Total EE. This argues against a genuine impact of perceived hrQoL on daily PA or vice versa, although correlation analyses are surely limited by small sample size. Considering this, trends seen for the correlation of HAQUAMS contentment with IPAQ Total duration, SWAmini active METs and vigorous PA as well as HAQUAMS total with SWAmini moderate PA and of HAQUAMS fatigue subscore with IPAQ moderate/vigorous PA and SWAmini mean METs and light PA deserve further investigation.

## Discussion

The amount of daily physical activity was reduced in PwMS compared to HC. This was expected from previous reports [28–30] as well as from the theoretical conceptualization of PA as the behavioral correlate of disability in MS [31, 32]. Given that the vast majority of evidence on PA in PwMS is from US cohorts, this is also the first confirmatory study in German MS subjects.

When measured objectively with SWAmini, group differences were seen for all parameters except light PA and were most pronounced for higher intensities, indicating the generally lower participation of PwMS in strenuous physical activities. The three most distinctive parameters were step count, mean METs and active METs. This supports the description of changes in PA as (1) reduction in global walking mobility and (2) reduced activity-related EE [7, 33, 34].

Effect sizes ranged from 0.63 to 0.86, which is comparable to previous reports of large MS cohorts [28, 35–37].

While daily step counts were similar to reports using other devices [36, 38, 39], the mean duration of MVPA activities observed in this study was higher than reported from US cohorts (MVPA < 23.5 and < 33.8 min/day in PwMS and HC) [35, 36] but lower than in other non-diseased populations [40]. Besides different recruitment bias and assessment devices, differences in activity lifestyle may also contribute.

An exploratory analysis of parameter inter-correlations (Additional file 2: Table S2) revealed that mean METs were most tightly associated with MVPA, active METs most strongly related to the amount of vigorous PA while step count showed only moderate correlations to both, mean METs and MVPA. Its lack of association with active METs and vigorous PA indicates that locomotor activity is predominantly performed within the low to moderate intensity segments.

This underlines that step count and EE capture complementary aspects of PA: subjects with gait or balance impairment may walk similar distances as HC but at lower speed (lower intensity) or – conversely - walk less efficiently, thus increasing the energy expended per step, which has indeed been shown in PwMS [41, 42].

Although our cohort included rather physically active PwMS, we observed a generally low amount of vigorous activities in PwMS with only one subject performing very vigorous PA, which impeded statistical comparison for this parameter. Concerning light activities, our results imply that this activity segment represents inevitable ADL activities, performed to a similar extent by HC and PwMS. It should further be noted that the group differences in PA relate to a segment of only about 10 min per hour, while both groups spend the largest part of their wake-time physically inactive. According to the MET definition of light PA, this may comprise low-intensity locomotor activity as well as sedentary time except sleeping which explains the seeming discrepancy to reports of increased sedentary time in PwMS [43, 44]. The effect of occupational status on PA was somewhat unexpected in its direction (tendency to even larger weekend – weekday difference in unemployed subjects) and small effect size, as others reported employment as a relevant factor for PA among PwMS [5, 35, 44, 45], however, in much larger cohorts. From our observation of a lower proportion of PwMS employed (Table 1), that even seems to decrease with higher EDSS scores (Additional file 3: Figure S1), we conclude that effects of occupational status and MS progression on daily PA may be difficult to distinguish or even counteract each other.

Group differences in PA could also be detected with the subjective IPAQ long form and were observed for the amount of MPA and MVPA, but not for the time spent walking. Convergent to SWAmini data, PwMS perceive themselves as (and according to SWAmini “are”) less engaged in higher intensity physical activities at group level. This is remarkable given the small group size and previous reports on a tendency for unfit (otherwise healthy) individuals to overestimate their participation in MVPA activities by up to 37% [46]. The questionnaire was easily applicable in our cohort and data exclusion or truncation rules had to be applied at similar rates in HC and PwMS.

With respect to IPAQ walking, it has to be considered, however, that IPAQ data refer only to reported bouts of over 10 min, although most locomotor activity, i.e. steps accumulated during the day, is likely to occur in shorter bouts. When relating results from subjective to objective assessment, we therefore subsumed reported walking activity within moderate PA.

The observed convergence for this matched parameter pair (r .507) was higher than expected from previous results obtained with other means of objective and subjective PA assessment in PwMS [36, 39, 47–49]. Similarly, the relation of IPAQ PA levels to selected SWAmini PA parameters roughly converge with commonly used cut-offs to detect low and highly active lifestyles by step count or mean METs (step count low level < 5000 steps/day, moderate level 5000–7500 steps/day and high level > 7500 steps/day; mean METs: very low level < 1.2 METs, low level 1.2–1.4 METs, moderate level 1.4–1.6 METs and high level > 1.7 METs) [23, 38]. This supports the validity of this easily applicable classification, e.g. for screening purposes. The trend seen for group difference (*p* = 0.15) is likely due to small sample size. Only one person with MS was classified as highly active, probably due to high IPAQ Walking durations despite a not specifically high step count around 7000/day and mean METs of 1.5.

This part of analysis further suggested that step count rather differentiates high from moderate PA levels, which may apply early in disease, while mean METs (related tightly to MVPA) is more suited to distinguish between moderate and low PA level. This further implies that increase in PA is mediated by different types of activity according to PA levels and that different PA parameters may be suited to monitor MS populations of different symptom severity.

Another aspect of IPAQ with relevance for intervention design is the context of PA. From our data, it seems unlikely that increases of PA in PwMS will be added as leisure/sports activity, whereas supporting active transportation or integrate PA into subjects’ lifestyle [50, 51] seem more appropriate. In this respect, it is worth re-considering and exploring the role of occupational status in daily PA and, specifically, active transportation. With regard to behavioral interventions, these probably need to be designed and evaluated specific to cultural settings according to activity lifestyle differences, for example between U.S. and German populations.

SWAmini parameter correlations to EDSS were moderate in magnitude, which is similar to previously reported coefficients between −.34 and −.70 [33, 39, 52–54]. Again, inspection of the parameter plots per EDSS score (Fig. 2) suggests reduced step count as an early feature in MS while decline in the daily amount of MVPA occurs beyond EDSS > 3, i.e. at stages with manifest decrease in walking mobility.

Corresponding IPAQ – EDSS plots revealed, that despite lower duration of MVPA in PwMS at group level, among those less severely affected, self-report of moderate activities was even higher than in HC. This paradoxical relation was even more pronounced for subjectively assessed walking duration and total EE. As discussed above, this may be interpreted as either a larger perceived exertion when physically active (despite unchanged or lower activity counts on “objective” assessment) or a systematic reporting bias in subjects aware of physical limitations, which has been reported in different populations [13]. Despite this and with only a small number of subjects remaining for IPAQ MPA and MVPA to EDSS analysis, correlations with symptom severity were similar to those observed with SWAmini. Further, IPAQ PA level assignment was related to EDSS scores. Both support the validity of IPAQ results in our PwMS cohort, although interpretations from individual absolute IPAQ results cannot be recommended.

With respect to HAQUAMS, we observed distributions of subscores similar to those reported in another German MS cohort [26] as well as expected correlations with EDSS.

However, the amount of daily moderate PA or daily step count were not related to HAQUAMS sum score, but seemed only related to the perceived impact of MS on lower limb function. This result was irrespective of the means of PA assessment (Tables 3 and 4) and adds to previous findings that PA in PwMS is only indirectly related to hrQoL, most likely mediated through physical function [55], although data on this topic are not abundant. One interventional study reported improved HAQUAMS total scores with intervention along with improvements in lower limb coordination [56]. Vice versa, decreased hrQoL as such seems unrelated to decrease in PA but related to a multitude of not directly disease-related factors [55, 57–61]. This implies, that interventions aiming to increase PA should target walking ability and physical limitations of the lower limbs, while the quantitative assessment of lower limb function may be a potentially useful surrogate of PA for intervention monitoring.

Our findings, in line with previous reports, support the notion that both objective and subjective PA assessment may be appropriate depending on the purpose and resources of the study [39], a major limitation of both being the inherent variability in PA that requires long-term recording over several days. Further, specific limitations of different devices - questionnaires or activity monitors - have been studied in different populations [18, 42, 62, 63] and rather point to an approximation of “true” PA from different perspectives instead of one golden assessment standard. Thus, individual PA parameters may rather be interpreted as reflecting a subject’s activity level than as meaningful quantitative parameters per se. Accordingly, clinically meaningful differences as determined for step count, for example, are expectedly large given the generally high standard deviations within groups of healthy subjects (here: 2397/day). It seems therefore justified to evaluate the validity of more amenable and standardized quantitative performance measures as predictors of PA in PwMS. Our data add evidence to previous findings that suggest measures related to walking and lower limb function as most promising candidates.

## Conclusion

Both methods of assessment seem applicable in PwMS and able to describe reductions in daily PA at group level. Whether they can be used to track individual effects of interventions to enhance PA levels needs further exploration. The relation of PA measures with hrQoL seen with lower limb mobility suggests lower limb function not only as a major target for intervention to increase PA but also as a possible surrogate for PA changes.

## Additional files

Additional file 1: Table S1. Overview of SenseWear® (SWAmini) and IPAQ (long version) parameters. (DOCX 14 kb)
Additional file 2: Table S2. Correlations between SWAmini parameters. (DOCX 13 kb)
Additional file 3: Figure S1. Relation of occupational status to EDSS. (DOCX 24 kb)
Additional file 4: Figure S2. Correlation of three parameters of physical activity derived from objective (SWAmini) and subjective (IPAQ) assessment after transformation into comparable units. (A) IPAQ Walking + MPA duration and SWAmini MPA duration (min/day), (B) IPAQ VPA duration and SWAmini VPA duration (min/day) and (C) IPAQ Total EE and SWAmini Active EE (MET*min/day). (DOCX 78 kb)

## Abbreviations

EDSS: Expanded Disability Status Scale
EE: Energy expenditure
HAQUAMS: Hamburg Quality of Life Questionnaire in Multiple Sclerosis
hrQoL: Health-related quality of life
IPAQ: International Physical Activity Questionnaire
LPA: Light physical activity
MET: Metabolic equivalents
MPA: Moderate physical activity
MVPA: Moderate-to-vigorous physical activity
PA: Daily physical activity
SWAmini: SenseWear mini® Armband
VPA: Vigorous physical activity
VVPA: Very vigorous physical activity
