# Supplementary material for: Subjective and objective assessment of physical activity in multiple sclerosis and their relation to health-related quality of life
Source: BMC Neurol. 2017 Jan 13;17:10. doi: 10.1186/s12883-016-0783-0 (PMC5237144; doi:10.1186/s12883-016-0783-0)
Supplement: Additional file 1: Table S1. — Overview of SenseWear® (SWAmini) and IPAQ (long version) parameters. (DOCX 14 kb) [file 12883_2016_783_MOESM1_ESM.docx]

**Additional file 1**

**Table S1: Overview of SenseWear® (SWAmini) and IPAQ (long version) parameters.**

The metabolic equivalent of task (MET) is an expression of energy expenditure as multitudes of estimated energy expenditure at rest (1 MET=1 kcal/hour/kg body weight). SWAmini data are calculated per hour/per day as the weighted mean of the 7-day recording period accounting for intra-week differences. IPAQ data are calculated per day from all reported activity for the 7-day period divided by seven.

| **Device** | **Parameter** | **Unit** | **Description** | **Interpretation** |
| --- | --- | --- | --- | --- |
| **SWAmini** | **Step count** | Steps/hour | Step count per hour | Gives an overview of all locomotor activity of the subject irrespective of intensity |
|  | **Mean METs** | MET | Mean of MET attributions of all 1-min episodes per day of recording time | Indicator of general activity levels, reflecting both, intensity and duration of physical activity throughout the day |
|  | **Active METs** | MET | Mean of all MET attributions from 1-min episodes of MET> 3 per day of recording time | Indicator of mean intensity of physical exertion |
|  | **Active Energy Expenditure** | MET*min/ day | Active METs multiplied by overall duration (min) of activity of MET>3 per day | Sum of all activity related energy expenditure (defined as MET>3) per day, reflecting both, intensity and duration, of physical activity |
|  | **LPA** | min/hour | Duration of all activity of MET 1.5-3 intensity | Duration of physical activity of low intensity |
|  | **MPA** | min/hour | Duration of all activity of MET 3-6 intensity | Duration of physical activity of moderate intensity |
|  | **VPA/VVPA** | min/hour | Duration of all activity of vigorous (MET 6-9, VPA) or very vigorous (MET>9, VVPA) intensity | Duration of physical activity of vigorous or very vigorous intensity |
| **IPAQ** | **Walking duration** | min/day | Daily time spent in walking activity (only bouts of >10 min) | Indicator of the individual’s locomotor activity |
|  | **MPA duration** | min/day | Daily time spent in moderate intensity activities (only bouts of >10 min) | Indicator of all moderate intensity activities except walking |
|  | **VPA duration** | min/day | Daily time spent in vigorous intensity activities (only bouts of >10 min) | Indicator of all vigorous intensity activities |
|  | **Total duration** | min/day | Time spent in any physical activity (only bouts of >10 min) | Duration of all physical activity (MVPA and walking) |
|  | **Total EE** | MET*min/day | Sum of all activity related energy expenditure (converted into MET estimates) multiplied by duration (min) of each activity and days with activity, divided by seven | Sum of all activity related energy expenditure per day, reflecting both, intensity and duration, of physical activity |
|  | **work, domestic, leisure, transportation EE** | MET*min/day | Sum of all activity related energy expenditure (including walking) per contextual domain | Context of energy expenditure |
|  | **low, moderate or high physical activity level** | - | Individual IPAQ result converted into one of three IPAQ categories according to user manual | IPAQ category of individual’s physical activity level |
