# Supplementary material for: Subjective and objective assessment of physical activity in multiple sclerosis and their relation to health-related quality of life
Source: BMC Neurol. 2017 Jan 13;17:10. doi: 10.1186/s12883-016-0783-0 (PMC5237144; doi:10.1186/s12883-016-0783-0)
Supplement: Additional file 2: Table S2. — Correlations between SWAmini parameters. (DOCX 13 kb) [file 12883_2016_783_MOESM2_ESM.docx]

**Additional file 2**

**Table S2. Correlations between SWAmini parameters.**

Correlations were performed for the whole group and reported as Pearson’s rho.

|  | Step count (per hour) | Mean METs (mean per day) | Active METs (mean per day) | Total EE (kcal/ hour) | Active EE (kcal/ hour) | LPA duration (min/ hour | MPA duration (min/ hour) | MVPA duration (min/ hour) |
| --- | --- | --- | --- | --- | --- | --- | --- | --- |
| Mean METs (mean per day) | **.570^**^** |  |  |  |  |  |  |  |
| Active METs (mean per day) | .165 | **.747^**^** |  |  |  |  |  |  |
| Total EE (kcal/hour) | **.310^*^** | **.636^**^** | **.338^*^** |  |  |  |  |  |
| Active EE (kcal/hour) | **.376^**^** | **.820^**^** | **.572^**^** | **.852^**^** |  |  |  |  |
| LPA duration (min/hour) | **-.279*** | -.198 | .084 | -.155 | -.020 |  |  |  |
| MPA duration (min/hour) | **.555^**^** | **.808^**^** | **.475^**^** | **.581^**^** | **.794^**^** | -.018 |  |  |
| MVPA duration (min/hour) | **.524^**^** | **.879^**^** | **.609^**^** | **.606^**^** | **.844^**^** | -.018 | **.972^**^** |  |
| VPA duration (min/hour) | .234 | **.768^**^** | **.788^**^** | **.454^**^** | **.681^**^** | -.013 | **.528^**^** | **.712^**^** |

Significance: * p-value < 0.05 and ** p-value <0.01.
