# Supplementary material for: Subjective and objective assessment of physical activity in multiple sclerosis and their relation to health-related quality of life
Source: BMC Neurol. 2017 Jan 13;17:10. doi: 10.1186/s12883-016-0783-0 (PMC5237144; doi:10.1186/s12883-016-0783-0)

**Additional file 3**

**Supplemental Figure 1: Relation of occupational status to EDSS.**

Subjects with employment are depicted in light blue, unemployed in dark blue.


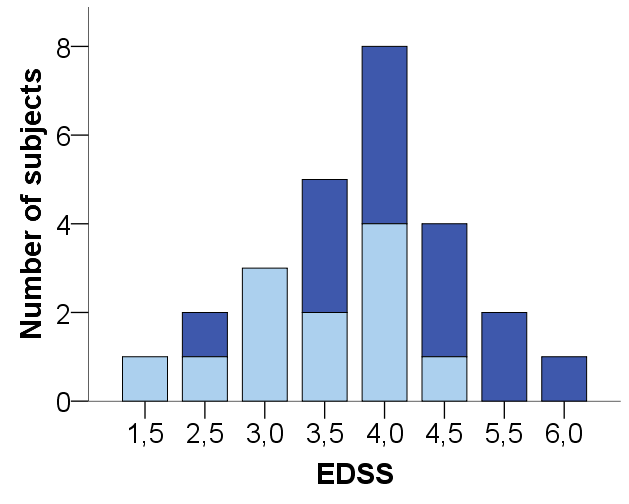

Supplement: Additional file 3: Figure S1. — Relation of occupational status to EDSS. (DOCX 24 kb) [file 12883_2016_783_MOESM3_ESM.docx]
