# Supplementary material for: Subjective and objective assessment of physical activity in multiple sclerosis and their relation to health-related quality of life
Source: BMC Neurol. 2017 Jan 13;17:10. doi: 10.1186/s12883-016-0783-0 (PMC5237144; doi:10.1186/s12883-016-0783-0)

**Additional file 4**

**Supplemental Figure 2: Correlation of three parameters of physical activity derived from objective (SWAmini) and subjective (IPAQ) assessment after transformation into comparable units. (A) IPAQ Walking+MPA duration and SWAmini MPA duration (min/day), (B) IPAQ VPA duration and SWAmini VPA duration (min/day) and (C) IPAQ Total EE and SWAmini Active EE (MET*min/day).**

Open circles denote HC subjects, filled circles denote PwMS. Please note that despite equal units the scaling of both axes is different for better readability.


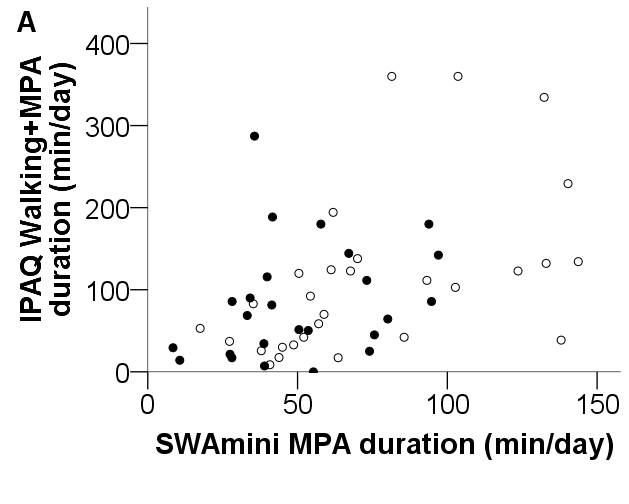


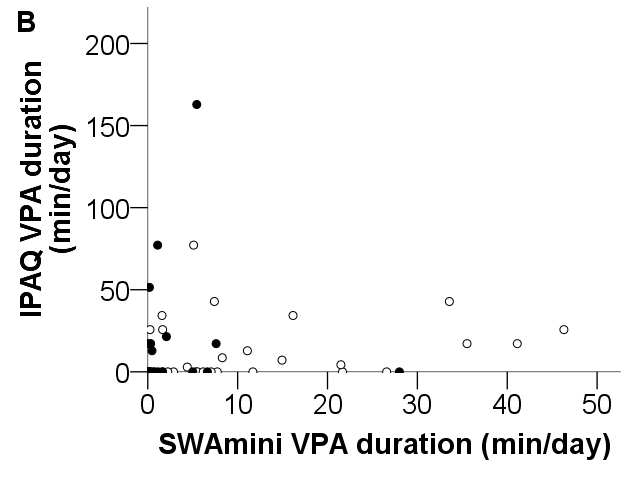


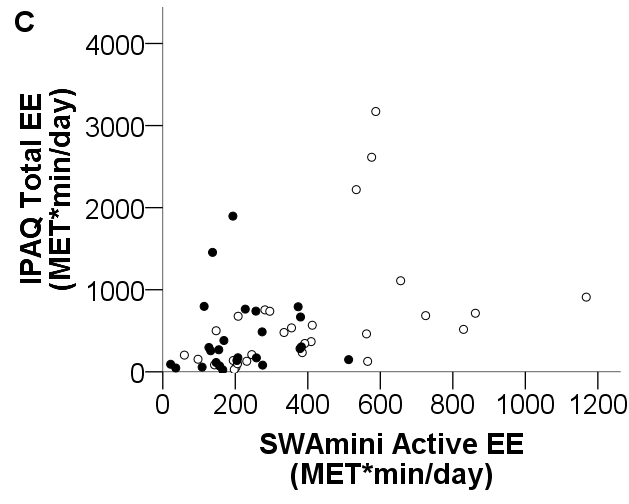

Supplement: Additional file 4: Figure S2. — Correlation of three parameters of physical activity derived from objective (SWAmini) and subjective (IPAQ) assessment after transformation into comparable units. (A) IPAQ Walking + MPA duration and SWAmini MPA duration (min/day), (B) IPAQ VPA duration and SWAmini VPA duration (min/day) and (C) IPAQ Total EE and SWAmini Active EE (MET*min/day). (DOCX 78 kb) [file 12883_2016_783_MOESM4_ESM.docx]
